# Evaluation of the applicability of the current CDC pediatric ventilator-associated events (PedVAE) surveillance definition in the neonatal intensive care unit population

**DOI:** 10.1186/s12887-022-03236-y

**Published:** 2022-04-07

**Authors:** Novisi Arthur, Ishminder Kaur, Alison J. Carey

**Affiliations:** 1 Division of Neonatology, St. Christopher’s Hospital for Children, Philadelphia, USA; 2 Division of Infectious Diseases, Department of Pediatrics, University of California Los Angeles, Los Angeles, USA; 3 Drexel University College of Medicine, Philadelphia, USA

**Keywords:** Neonate, Neonatal intensive care unit, Pediatric ventilator-associated events, Mechanical ventilation

## Abstract

**Background:**

There is limited data on pediatric ventilator-associated events (PedVAE) in the neonatal intensive care unit (NICU) setting, since the CDC mandated state reporting of these events in January 2019. This study sought to describe PedVAE rates and characteristics in the NICU population.

**Methods:**

Single-center case-control study of infants requiring mechanical ventilation in a 39-bed level IV NICU between January 1, 2018 and December 31, 2020. Baseline infant demographic, respiratory support and antibiotic use data was obtained and comparisons were performed between patients with potential PedVAEs and those without events.

**Result:**

Two hundred and nine infants were mechanically ventilated. Two of the 126 patients ventilated for ≥4 days met CDC criteria for PedVAEs with a total of 3 events, and 32 (25%) received antibiotics with escalation of respiratory support, primarily for tracheitis.

**Conclusion:**

NICU-specific data on PedVAE is limited. Only 2 infants in the study period met the current CDC criteria for PedVAE with a rate of 0.9 events per 1000 ventilator days. The current CDC PedVAE definition might be inadequate to identify actionable VAEs to inform prevention efforts in the NICU population, and alternate indices could better characterize these events.

## Introduction

Mechanical ventilation is an essential, life-saving therapy for infants with critical illness and respiratory failure [1]. However, mechanically ventilated neonates are at high risk for complications including bronchopulmonary dysplasia (BPD), ventilator-associated pneumonia (VAP), septicemia, pulmonary edema, and acute respiratory distress syndrome, which can prolong the duration of mechanical ventilation, length of hospitalization, and contribute significantly to neonatal morbidity and mortality [2, 3]. Surveillance for ventilator-associated events (VAEs) is a critical component of patient safety and quality improvement initiatives in health care facilities and is an integral part of the safety surveillance systems managed by the Centers for Disease Control and Prevention (CDC) [4].

Traditionally, surveillance for VAEs was limited to VAP alone, but the most widely accepted VAP definitions are neither sensitive nor specific, due to the subjectivity of clinical and chest radiographic findings of pneumonia, key requirements of the CDC’s VAP definition [5, 6]. The lack of a precise, reproducible and clinically meaningful definition has implications for assessing effectiveness of prevention strategies. In 2013, the CDC introduced the VAE surveillance definition to address these limitations in the adult populations [7]. These new definitions eliminated the subjectivity and variability associated with diagnosing VAP and broadened the range of surveillance to include both infectious and non-infectious etiologies associated with deterioration on the ventilator.

VAE surveillance was initially not used in neonatal and pediatric locations based on recommendations of a separate working group the CDC organized in 2012 [7]. Subsequently, pediatric and neonatal adaptations for VAE surveillance criteria were defined based on increase in the fraction of inspired oxygen (FiO_2_) and/or mean airway pressure (MAP) after a period of stability or improvement on the ventilator [8, 9].

In a retrospective cohort study involving adult patients, VAEs were associated with more days to extubation, more days to hospital discharge, and higher mortality risk [10]. In addition, antimicrobial use is common in pediatric ventilator associated conditions, which include both infectious and non-infectious complications [10, 11]. PedVAE is a surveillance definition and is not intended for use in clinical management of infants. State-mandated reporting of PedVAE started in 2019 and is not standardized. Research on pediatric VAEs focuses on the pediatric intensive care unit (PICU) setting [4, 6]. Therefore, epidemiology of PedVAE in the neonatal intensive care unit (NICU) is limited. We sought to describe PedVAE rates and event characteristics in the NICU population from a single 39-bed level IV NICU before and after the implementation of the state-mandated reporting of CDC PedVAE surveillance definition in January 2019. In addition, risk factors to develop PedVAEs were determined. Finally, we analyzed infants who were mechanically ventilated for at least 4 days and had a new antibiotic treatment course initiated during the time of mechanical ventilation as a surrogate marker of events that require quality improvement interventions.

## Methods

### Study site

St. Christopher’s Hospital for Children’s NICU, a 39-bed Level IV regional perinatal center, is located in Philadelphia, PA. Nearly 250 neonates are admitted annually; all patients are out-born and transferred in from regional NICUs. Admitted infants frequently require advanced surgical and pediatric subspecialty services.

### Study design

A database of admissions to the NICU is maintained by the Division of Neonatology for reporting purposes. All admissions from January 1, 2018 to December 31, 2020 were screened for use of mechanical ventilation. This single center, case-control study was conducted with a detailed retrospective chart review of these mechanically ventilated patients. Inclusion criterion was any patient in the NICU receiving mechanical ventilation via endotracheal tube or tracheostomy. Patients on extracorporeal membrane oxygenation were excluded from PedVAE surveillance for the duration of time when this support was in place, based on the CDC definition of PedVAE.

### Human subjects accordance statement

This study was reviewed by the Institutional Review Board at Drexel University College of Medicine and was determined to be exempt (protocol: 190500718). The need for informed consent was waived as this was deemed exempt human subjects research. All methods were carried out in accordance with relevant guidelines, and the information was recorded by the investigator in such a manner that subjects could not be identified, directly or through identifiers linked to the subjects.

### Definitions [12]

Patients needed to be mechanically ventilated for at least 4 calendar days to fulfill PedVAE criteria. PedVAEs are defined by a 14-day period, starting on the day of onset of worsening oxygenation (the event day, Day 1). A new PedVAE cannot be identified or reported until this 14-day period has elapsed [1]. The patient must have a baseline period of stability or improvement on the ventilator, defined by ≥2 calendar days of stable or decreasing daily minimum FiO_2_ or MAP values. The baseline period is defined as the two calendar days immediately preceding the first day of increased daily minimum MAP or FiO_2_. The daily minimum FiO_2_ is defined as the lowest value of FiO_2_ documented during a calendar day that is maintained for more than 1 h. Daily minimum MAP is the lowest value documented during the calendar day. For patients less than 30 days old, daily minimum MAP values 0–8 cmH_2_O are considered equal to 8 cmH_2_O for the purposes of surveillance. For patients ≥30 days old, daily minimum MAP values 0–10 cmH_2_O are considered equal to 10 cmH_2_O for the purposes of surveillance.

A PedVAE is defined if after a period of stablity or improvement on the ventilator, the patient has at least one of the following indicators of worsening oxygenation: 1. Increase in the daily minimum FiO_2_ of ≥25% over the daily minimum FiO_2_ of the first day in the baseline period, sustained for ≥2 calendar days; or 2. Increase in the daily minimum MAP values of ≥4 cmH_2_O over the daily minimum MAP of the first day in the baseline period, sustained for ≥2 calendar days.

Additionally, infants who were mechanically ventilated for at least 4 days and had a new antibiotic treatment course initiated during the time of mechanical ventilation were analyzed for worsening oxygenation. We coined these events as potential PedVAEs for the purpose of this study to identify another surrogate marker of events that require quality improvement interventions.

### Statistical analysis

All statistical analysis for this research was conducted using SPSS version 25.0. Descriptive data were reported for categorical variables using either count or percent for each category, and for continuous data mean with standard deviation. Comparisons between potential PedVAE/PedVAE cases (*n* = 32) and the No-Event cohort (*n* = 94) were conducted using chi-square test of association for categorical variables and group t-test for continuous variables.

A logistic regression was used to determine if potential PedVAE/PedVAE cases could be explained by the duration of mechanical ventilation, gestational age (measured in weeks) using greater than 32 as a reference, and birth weight measured categorically with greater than 2500 g as the reference. A reasonable sample size for a logistic regression model is at least 10 events per independent variable. For our study, we had 32 patients with a PedVAE/potential PedVAE. Therefore, three independent variables were included in the logistic regression. Reporting on the logistic regression included beta coefficient, *p*-value of the beta coefficient, odds ratio, and 95% confidence interval of the odds ratio. The logistic regression was performed using the ‘Enter’ method, with no forward or backward elimination selected because of the small sample size, and to hold iterations of the model at a minimum.

Due to the exploratory nature of this analysis and alpha level of 0.05 (*p* < 0.05) was used to determine significance for all tests. No corrections were applied to the data for multiple comparisons, and no missing value imputations were performed.

## Results

### Baseline characteristics of the study population

**Fig. 1:**
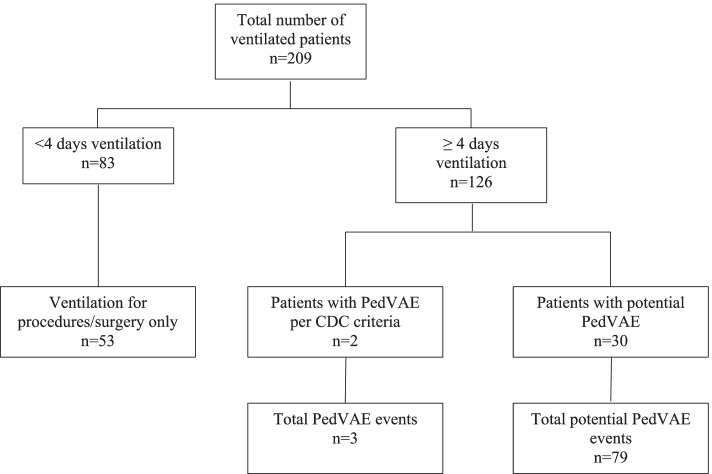
Ventilated Patients in the NICU

A total of 209 patients were ventilated in the Level IV NICU out of 676 admissions between January 1, 2018 and December 31, 2020. Eighty-three (40%) were excluded because they were ventilated for less than 4 days (Fig. 1). Of these 83 infants, 53 (64%) were intubated for procedures only. One hundred and twenty-six patients (126/209; 60%) were intubated for at least 4 days and comprised the study population for PedVAE evaluation. Fifty-seven of these 126 patients (45%) were < 1000 g at birth, and 58 out of 126 (46%) were born at less than 28 weeks gestation.

### Application of the CDC PedVAE definition to the NICU population

Of the 126 infants eligible for evaluation, only 2 patients met the CDC criteria for PedVAE during the 3-year study period. Both patients who developed PedVAE based on the CDC criteria were 25-week gestation infants, and Hispanic in origin. The first patient was male and developed a PedVAE on day of life (DOL) 40 (day 35 of mechanical ventilation) based on a sustained increase in the daily minimum FiO_2_ by 32%. The second patient was a female who developed 2 PedVAEs during her NICU stay. The first event occurred on DOL 51 (day 33 of mechanical ventilation) based on a sustained increase in her daily minimum MAP by 4 cmH_2_0, and the second event occurred on DOL 181 (day 107 of mechanical ventilation) based on another sustained increase in her daily minimum MAP by 5 cmH_2_0.

In all three of these events, antibiotics were prescribed by the primary medical team. The first patient was 1 day post-operative from an exploratory laparotomy for necrotizing enterocolitis and had blood culture-proven sepsis with *Enterococcus faecalis.* He ultimately succumbed to the infection within 24 h of the PedVAE. The two events in the second patient were treated for culture-negative sepsis for 7–14 days.

Upon further review of the cases where there was an escalation of respiratory support, but the event did not meet CDC criteria for a PedVAE, antibiotics were prescribed often. We next analyzed the 126 patients who were mechanically ventilated for more than 4 days and there was initiation of a new antibiotic course. Using this as a surrogate marker for a clinical deterioration while mechanically ventilated, respiratory support parameters were evaluated when antibiotics were initiated and an additional 30 patients with potential PedVAE were identified (Fig. 1). There were 79 events recorded among 30 patients with potential PedVAEs. The total number of ventilator days for the 32 patients was 3312.

### Characteristics of infants with PedVAE/potential PedVAE

We sought to describe the clinical characteristics of those infants who had an event and those who did not. Infants with potential PedVAE/PedVAE were more preterm (*p* < 0.001), had a lower birth weight (*p* < 0.001), spent a longer duration on the ventilator (*p* < 0.001) and had a longer NICU stay (*p* < 0.001) compared to those who did not have potential PedVAE/PedVAE. They were also more likely to be small for gestational age and have other complications of prematurity including respiratory distress syndrome, bronchopulmonary dysplasia, patent ductus arteriosus and retinopathy of prematurity (Table 1). Therefore, those patients who had an event were more likely to be preterm and therefore had many characteristics associated with prematurity.

**Table 1:** Neonatal and Maternal Characteristics

Maternal/Neonatal Characteristic	No Event (***n*** = 94)	Potential PedVAE/PedVAE (***n*** = 32)	***P***-value
Maternal Age (mean, years)	29.7 ± 6.4	27.4 ± 6	0.083
Gestational Age (mean, weeks)	32.1 ± 6.3	27.5 ± 4.6	< 0.001
< 28 weeks	35 (37.2%)	23 (71.9%)	< 0.001
28–32 weeks	8 (8.5%)	4 (12.5%)	
> 32 weeks	51 (54.3%)	5 (15.6%)	
Birth Weight (mean, grams)	1166	745	0.006
< 1000 g	34 (36.2%)	23 (71.9%)	0.001
1000 – 2500 g	20 (21.3%)	5 (15.6%)	
> 2500 g	40 (42.6%)	4 (12.5%)	
Age on Admission (mean, days)	11.8	28.3	0.013
Duration of Mechanical Ventilation (mean, days)	19.4	114.78	< 0.001
Length of NICU stay (mean, days)	77.5	166.5	< 0.001
Prenatal Steroids (%)	36	50	0.210
Prenatal Antibiotics (%)	24	34	0.275
Hispanic (%)	36	28	0.407
Female (%)	46	44	0.845
SGA (%)	11	31	0.006
Intubation in Delivery Room (%)	50	66	0.126
Surfactant (%)	54	81	0.007
RDS (%)	44	75	0.002
Caffeine (%)	45	81	< 0.001
Early Onset Sepsis (%)	1	0	0.558
Late Onset Sepsis (%)	12	9	0.717
Retinopathy of Prematurity (%)	37	75	< 0.001
Patent Ductus Arteriosus (%)	56	78	0.012
Bronchopulmonary Dysplasia (%)	31	78	< 0.001
BPD-Pulmonary Hypertension (%)	4	28	< 0.001
Intraventricular Hemorrhage (%)	29	34	0.547
Necrotizing Enterocolitis (%)	3	38	0.060
Death (%)	16	13	0.779

### Increased ventilator duration is associated with potential PedVAE/PedVAE

To determine the clinical factors with the greatest biologic plausibility in terms of predisposing to PedVAE, we performed a logistic regression. Although there were several clinical characteristics which were more frequent in the potential PedVAE/PedVAE group, these clinical characteristics are also much more common in the extremely preterm population. Based on the number of events in our population (32), we analyzed three variables which were likely to be driving these events: gestational age, birth weight and duration of mechanical ventilation. When birth weight and gestational age were controlled for, there was no longer a significant association with potential PedVAE/PedVAE. However, increased ventilator duration remained significant, with higher duration demonstrating a risk factor with a slightly elevated odds ratio equal to 1.030 (Table 2**:** 95% confidence interval 1.013–1.047).

**Table 2:** Logistic Regression Showing Association between Potential PedVAE/PedVAE and Duration of Mechanical Ventilation, Gestational Age and Birth Weight

Variable	Category	beta	***P***-value	Odds Ratio	95% Confidence Interval	
					Lower	Upper
Duration of Mechanical Ventilation (days)		0.030	< 0.001	1.030	1.013	1.047
Gestational Age (weeks)	< 28	0.637	0.587	1.991	0.190	18.804
	28–32	1.201	0.395	3.323	0.379	29.136
	> 32	Reference	N/A			
Birth weight (g)	< 1000	0.718	0.568	2.049	0.175	24.008
	1000–2500	0.640	0.537	1.896	0.249	14.451
	> 2500	Reference	N/A			

### Infection characteristics of infants with potential PedVAEs

**Fig. 2:**
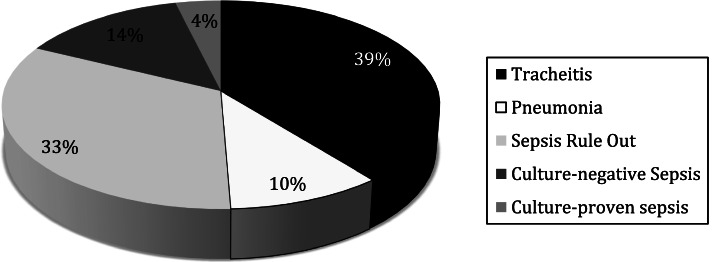
Indication for New Antibiotic Use

Of the 79 events recorded among 30 patients with potential PedVAEs, 3 (4%) were treated for blood culture-proven sepsis. Two patients were treated for *Staphylococcus epidermidis* and one patient was treated for *Candida albicans.* Eleven out of 79 events (14%) were treated for culture-negative sepsis for 7 to 10 days, and in 26 out of 79 (33%) antibiotics were prescribed for 48–72 h for sepsis rule out (Fig. 2).

The most common indication for antibiotic use was tracheitis (Fig. 2) comprising 31 out of 79 events (39%). The most commonly isolated organisms from tracheal aspirate cultures were: *Pseudomonas aeruginosa* (12/31; 39%), followed by *Klebsiella pneumoniae* (6/31; 19%) and *Staphylococcus aureus* (4/31; 13%). In 8 out of 79 events (10%), patients were treated for pneumonia. In addition to chest radiograph findings, tracheal aspirate cultures associated with these 8 events showed the following: cytomegalovirus (CMV pneumonitis in an infant with reactivation, antibiotics given prior to CMV confirmation), *Staphylococcus aureus* (with a concern for aspiration), *Pseudomonas aeruginosa* (2 events), methicillin-resistant *Staphylococcus aureus* (MRSA), *Escherichia coli*, *Serratia marcescens* and *Klebsiella pneumoniae*. These patients did not meet CDC criteria for ventilator-associated pneumonia.

### Modification of the CDC definition

Our review revealed that there were 3 PedVAE events in a 3-year period, using the current CDC criteria. Infants frequently failed to meet the criteria because of the stipulation for a period of stability in oxygen requirement or ventilator settings. We questioned how a modification of the criteria might impact the number of identified events. First, events were evaluated without the requirement for stable or decreasing FiO_2_/MAP prior to the event, as well as the sustained increase for two or more calendar days following the event. Subsequently, the thresholds of the daily minimum increase in FiO_2_ were reduced in 5 % increments from the CDC guideline of 25% (Table 3). The window of a sustained increase was narrowed from 2 calendar days to 1 calendar day. Not surprisingly, the number of events increased as the FiO_2_ threshold was decreased, although there was no difference in the number of events with a threshold of 10% versus 15%. We similarly analyzed the events using a reduced MAP threshold and found there were only 2 events based on reduction in MAP threshold to 2. Taken together, with significant alteration of the CDC definition for a PedVAE, there are very few ventilator-associated events in the NICU population.

**Table 3:** Number of Potential PedVAE with Lower Thresholds for Daily Minimum Increase in FiO_2_ and MAP

FiO_**2**_ (%)	Number of Potential PedVAE	MAP (cmH_**2**_0)	Number of Potential PedVAE
≥ 20	4	≥3	0
≥15	8	≥2	2
≥10	8		
≥5	24		

## Discussion

NICU-specific PedVAE data are limited, with most studies of PedVAE occurring in the PICU or other acute care settings such as the CICU. In this single-center case-control study of ventilated infants, 2 patients met the PedVAE surveillance definition based on the CDC criteria with a total of 3 events. The PedVAE definition is based on an increase in the daily minimum FiO_2_ by ≥25% or MAP by ≥4 cmH_2_0 after a 2-day period of stability on the ventilator. Ventilator-associated conditions specific to pediatrics have been defined based on modification of the adult definition to use MAP instead of positive end-expiratory pressure (PEEP) [8]. PEEP can be difficult to measure in pediatric high frequency ventilator modes, and MAP may better represent lung compliance in the neonatal and pediatric population [8].

In the adult population, most VAEs are because of pneumonia, pulmonary edema, atelectasis or acute respiratory distress syndrome [10]. The low incidence of PedVAE in our study population could be attributed to the intrinsic differences in respiratory physiology and ventilation management strategies between adults and children. For example, patients in the NICU are more likely to be ventilated due to prematurity or bronchopulmonary dysplasia, as opposed to pneumonia or pulmonary edema.

Although many patients had episodes of significant FiO_2_ increase on the ventilator, the event did not qualify because the definition is based on the daily minimum value for the day. Other reasons for exclusion included no 2-day period of stability or decreasing FiO_2_ prior to the increase. This was evidenced by an increase in the number of potential events once the period of stability or decrease was removed. Different thresholds of fraction of inspired oxygen (FiO_2_) and PEEP can result in increased sensitivity and better correlation between the VAE definition in children and clinical outcomes in ventilated critically ill children [13].

An increase in MAP of 4 cmH_2_0 in the NICU population is very unlikely unless the patient is being transitioned from the conventional ventilator to high frequency oscillatory ventilation, which was the case in the patient who developed 2 PedVAEs based on the increase in her MAP. There were 2 potential events when the period of stability or decrease was removed and the threshold for increase in MAP was reduced to 2 cmH_2_0.

In the NICU population, infants who have a clinical deterioration and require increased ventilator settings frequently are initiated on empiric antibiotics and a blood culture is obtained to rule out sepsis. Therefore, initiation of a new course of antibiotics while mechanically ventilated was used to identify an additional 30 patients with 79 potential PedVAEs. There is poor correlation between clinician diagnosed ventilator-associated infection in PICUs and the proposed PedVAE definition [14].

Out of these 79 events, only 3 events (4%) had culture-proven sepsis with *Staphylococcus epidermidis* in 2 events and *Candida albicans* in 1 event. The primary indication for antibiotic use in our study population was tracheitis and the most common organism isolated in tracheal aspirate cultures was *Pseudomonas aeruginosa*. Similarly, 60% of tracheal aspirates in a Canadian PICU grew *Haemophilus influenzae* and *Pseudomonas aeruginosa* [15]. Antibiotic need and duration for ventilator-associated tracheitis in the pediatric population are not well defined [16, 17].

Patients with potential PedVAE/PedVAE were more likely to be born preterm, and there was a statistically significant increase in complications of prematurity such as respiratory distress syndrome (RDS), bronchopulmonary dysplasia (BPD) and retinopathy of prematurity in this population. Of note, patients with potential PedVAE/ PedVAE were admitted at an older age compared to those without PedVAE. This study was conducted at a Level IV NICU with all admitted infants transferred from peripheral Level II and III NICUs. Therefore, these patients have usually had complicated hospital courses at the referral sites, which culminates in transfer.

Age-specific comorbidities such as RDS, BPD and necrotizing enterocolitis in neonates and infants can independently result in prolonged duration of mechanical ventilation, and complicate clinical and laboratory diagnostic criteria of VAP [18]. To remove these confounding factors, a logistic regression was performed to determine if potential PedVAE/PedVAE cases could be explained by the duration of mechanical ventilation, gestational age, and birth weight. This showed a significant association between potential PedVAE/PedVAE and the duration of mechanical ventilation, which is consistent with the adult literature [10].

The rate of PedVAE in our study was 0.9 per 1000 ventilator days, and thus difficult to assess the attributable morbidity and mortality in our patient population. Only 2 infants met current PedVAE criteria in the 3-year study period. However, about one-quarter of ventilated infants received multiple sepsis evaluations and antibiotic courses associated with increased ventilator settings. The current PedVAE definition offers little guidance relating to antibiotic use, and might be inadequate to identify actionable VAE events to inform prevention efforts in the NICU population. To the best of our knowledge, there have been no studies on PedVAE in the neonatal population since the implementation of mandatory reporting of these events by the CDC in January 2019. Screening daily ventilator settings is feasible and efficient, however, this study reveals the challenges with the current surveillance definition and the need to identify alternate indices which could better characterize these events in the NICU population and predict adverse outcomes.

## Data Availability

The datasets generated and/or analysed during the current study are available in the BioStudies repository, https://www.ebi.ac.uk/biostudies/studies/S-BSST798.

## Abbreviations

CDC: Centers for disease control and prevention
VAP: Ventilator-associated pneumonia
VAE: Ventilator-associated event
PedVAE: Pediatric ventilator-associated event
NICU: Neonatal intensive care unit
BPD: Bronchopulmonary dysplasia
MAP: Mean airway pressure
FiO2: Fraction of inspired oxygen
PEEP: Positive end expiratory pressure
RDS: Respiratory distress syndrome
ROP: Retinopathy of prematurity
DOL: Day of life
